# Melting Enhancement in a Triple-Tube Latent Heat Storage System with Sloped Fins

**DOI:** 10.3390/nano11113153

**Published:** 2021-11-22

**Authors:** Mustafa Z. Mahmoud, Hayder I. Mohammed, Jasim M. Mahdi, Dmitry Olegovich Bokov, Nidhal Ben Khedher, Naif Khalaf Alshammari, Pouyan Talebizadehsardari, Wahiba Yaïci

**Affiliations:** 1 Radiology and Medical Imaging Department, College of Applied Medical Sciences, Prince Sattam Bin Abdulaziz University, Al-Kharj 16244, Saudi Arabia; 2 Faculty of Health, University of Canberra, Canberra 2601, Australia; 3 Department of Physics, College of Education, University of Garmian, Kurdistan, Kalar 46021, Iraq; hayder.i.mohammad@garmian.edu.krd; 4 Department of Energy Engineering, University of Baghdad, Baghdad 10071, Iraq; jasim@siu.edu; 5 Institute of Pharmacy, Sechenov First Moscow State Medical University, 8 Trubetskaya St., bldg. 2, 119991 Moscow, Russia; fmmsu@mail.ru; 6 Department of Mechanical Engineering, College of Engineering, University of Ha’il, Ha’il 53962, Saudi Arabia; N.khedher@uoh.edu.sa (N.B.K.); naif.alshammari@uoh.edu.sa (N.K.A.); 7 Laboratory of Thermal and Energetic Systems Studies (LESTE), National School of Engineering of Monastir, University of Monastir, Monastir 5000, Tunisia; 8 Centre for Sustainable Energy Use in Food Chains, Institute of Energy Futures, Brunel University London, Kingston Lane, Uxbridge UB8 3PH, UK; 9 CanmetENERGY Research Centre, Natural Resources Canada, 1 Haanel Drive, Ottawa, ON K1A 1M1, Canada

**Keywords:** melting, heat-transfer enhancement, phase-change material, sloped fins, triple-pipe heat exchanger

## Abstract

Due to the potential cost saving and minimal temperature stratification, the energy storage based on phase-change materials (PCMs) can be a reliable approach for decoupling energy demand from immediate supply availability. However, due to their high heat resistance, these materials necessitate the introduction of enhancing additives, such as expanded surfaces and fins, to enable their deployment in more widespread thermal and energy storage applications. This study reports on how circular fins with staggered distribution and variable orientations can be employed for addressing the low thermal response rates in a PCM (Paraffin RT-35) triple-tube heat exchanger consisting of two heat-transfer fluids flow in opposites directions through the inner and the outer tubes. Various configurations, dimensions, and orientations of the circular fins at different flow conditions of the heat-transfer fluid were numerically examined and optimized using an experimentally validated computational fluid-dynamic model. The results show that the melting rate, compared with the base case of finless, can be improved by 88% and the heat charging rate by 34%, when the fin orientation is downward–upward along the left side and the right side of the PCM shell. The results also show that there is a benefit if longer fins with smaller thicknesses are adopted in the vertical direction of the storage unit. This benefit helps natural convection to play a greater role, resulting in higher melting rates. Changing the fins’ dimensions from (thickness × length) 2 × 7.071 mm^2^ to 0.55 × 25.76 mm^2^ decreases the melting time by 22% and increases the heat charging rate by 9.6%. This study has also confirmed the importance of selecting the suitable values of Reynolds numbers and the inlet temperatures of the heat-transfer fluid for optimizing the melting enhancement potential of circular fins with downward–upward fin orientations.

## 1. Introduction

The cleaner production of energy in line with the switch to more sustainable energy sources, such as solar and wind, is widely acknowledged today as the primary approach for bringing the global energy system into a green and sustainable economy. However, energy from these sources is inherently intermittent, and conserving continuous and consistent power production requires the development and adoption of sophisticated storage schemes [1]. An efficient approach that has the potential to play a key role in increasing renewable energy utilization is thermal energy storage (TES) [2]. This approach may deal with any potential imbalance in energy supply and demand by storing the energy in thermal form for later use. Due to this, there is a growing interest to modify and improve the design of TES systems so that they can deliver remarkable cost savings for the different renewable energy applications. There are three primary methods of TES: sensible, latent, and thermochemical. Due to the relatively lower volume/mass requirements of the storage material and the almost no-swing temperature when storing/releasing the stored energy, latent TES is favored over the other two methods [3,4]. The widely known phase-changing materials (PCMs) are used as heat storage materials in latent TES systems. These materials have the ability to re-form their phase responding to solid–liquid transition processes. Thermal diffusivity and heat conductivity play a major role in identifying the usefulness of PCMs as TES materials, as well as their melting and solidification points, specific heat capacities, and latent enthalpies of fusion. As a result, the last few years have seen an increasing amount of research on modifying these properties to eliminate any difficulties or inconsistencies that may be inherent in the application of this TES technology.

One major inconsistency that most PCMs are affected by is their intrinsic poor heat conductivities, which poses a detrimental effect on the TES system’s response rates to the periodic energy storage/retrieval activities. Previous research identifies two major steps for overcoming this problem: (i) employing an adequate heat-transfer enhancer such as metal/graphite matrix, honeycomb, fin, nano-encapsulated phase-change materials (NEPCMs) [5,6,7], and heat pipe structures and (ii) using an efficient casing design to house the PCM so that a superior heat communication between the HTF and the PCM can be achieved [8,9]. Modifying the casing design by attaching fins to the thermally active walls is regarded as one of the most efficient approaches for intensifying the thermal responsiveness of energy storage systems [10,11,12,13,14]. When fins are properly designed, the high enhancement ratio, low manufacturing costs, and ease of installation are only a few of the benefits that can be attained [15]. As a result, the interest and amount of research on improving the configuration, dimensions, arrangement, and material utilization of fins are increasingly growing [16]. These parameters may be optimized depending on the type of the target application, the type of the PCM used in the system, and the required energy storage/recovery rates [17].

Fins of longitudinal, rectangular, triangular, pin-styled, and circular (annular) configurations have been the most widely investigated types in prior studies [18]. The circular fin features a straightforward design that promises high effectiveness for a minimum volume, particularly in the cylindrical PCM confinement vessels, making it of special relevance to this research [19]. Lacroix [20] investigated the presence of fins in the shell-and-tube storage unit and reported that circular fins were most efficient for the heat-transfer fluid (HTF) at low intake temperatures (ΔT ~ 5 K) and moderate flow rates ( $\dot{m}$ ≤ 0.015 kg/s). Kozak et al. [21] reported on the close-contact melting in a vertical shell-and-tube unit with circular fins and demonstrated that the existence of circular fins can play a far more essential role than just serving as heat-transfer enhancement surfaces. Mat et al. [22] demonstrated that adding circular fins not only serves the improvement of the overall system’s storage efficiency but also serves a more uniform temperature distribution in the PCM, which reduces the unwelcome thermal stress in the system. Yang et al. [23] found that including circular fins into PCM can enhance heat conduction and local natural convection, resulting in an almost 65% quicker melting rate in the vertical shell-and-tube TES unit. Jannesari and Abdollahi [24] added thin rings and circular fins into an ice-on-coil system to enhance the ice formation rate by 21% and 34% higher concerning the bare coil, respectively. Pathak et al. [25] added a magnetic fluid layer to improve the circular fin efficiency by 20% in a PCM triplex-tube heat exchanger. Kalapala and Devanuri [26] compared the performance of a circular-fin heat exchanger in the vertical and horizontal orientations and concluded that vertical configuration was performing better in terms of Nusselt number, PCM temperature, and melting rate except energy efficiency.

Recently, another strategy has been proposed for improving the enhancement potential of circular fins by applying nonuniformly fin distribution. Singh et al. [27] showed that employing circular fins with a nonuniform fin height along the HTF flow direction can improve the PCM charging rate by a maximum of 43%. Yang et al. [28] found that including circular fins of nonuniform design on fin position and pitch can facilitate more melting uniformity by about 87% in the vertical triplex-tube heat exchanger TES systems. However, Shahsavar et al. [29] reported that using a uniform distribution of circular fins on fin diameter and thickness is better than the nonuniform distribution from the perspective of melting and solidification in the vertical double-pipe TES systems. Tiari et al. [30] found that the gradual decrease in the circular fin height along the vertical direction was better for discharging alone; however, using uniform fin height was the best configuration for both charging and discharging. Zhu et al. [31] showed that the melting and solidifying growth rates can be improved by as much as 29.9 and 15.8%, respectively, when the end-side fin length of the circular fins increases at the bottom, which also helps reduce the local heat resistance.

The use of circular fins in conjunction with a triple-tube heat exchanger (TTHE) can address the disputes of low heat conductivities that most PCM-based storage systems suffer from. Compared with the cylindrical TES systems, the TTHE as a containment design exhibits a relatively large heat exchange area, which facilitates a good heat diffusion into PCM (RT-35) from the circulating HTF. This can also indirectly lower the thermal resistance of the PCM, allowing for more efficient thermal energy storage and retrieval. The primary objective of this research was to investigate the prospect of enhancing the PCM solidification in a new vertical TTHE arrangement with sloped circular fins. In this arrangement, the HTF flows in two opposing directions: the gravity direction in the inner tube and the opposite direction outside the middle tube of the TTHE system. This arrangement would support a better overall heat-transfer rate from the perspective of natural convection and thermal conduction. An additional objective was to analyze the effects of mass flow rate and temperature of heat-transfer fluid, as well as the distribution of circular fins and their orientation on the PCM thermal response rates during the energy discharging mode. It was also emphasized to evaluate the thermal response of PCM, while using inline and staggered fin arrangements between the inner tube and outer tube of the TTHE system based on the melting time and energy charging rate. To the best of the authors’ knowledge, there has been no prior study on the application of sloped fins with different fin sizes and orientations in PCM-based storage systems. Results are expected to provide remarkable advancements in the area of enhanced heat transfer and time saving in energy storage and retrieval modes.

## 2. Problem Description

A triple-pipe heat exchanger with sloped fins is investigated in this study, where circular fins are connected to the inner and outer pipes in the PCM domain. The PCM is placed in the middle pipe, while water as the heat-transfer fluid is passed through the inner and outer pipes to melt the PCM. The proposed system is compared with the case with uniform fins and finless. The length of the system is 250 mm, while the inner, middle, and outer diameters of the pipes are 20, 40, and 60 mm, respectively. The thickness of the tube is also considered 1 mm made from copper. The HTF is passed opposite to the gravity direction in the inner pipe while it is in the gravity direction in the outer tube, as established in the literature with higher performance compared with co-current directions for the fluid flow. Thus, the flow of the HTF in the heat exchanger is counter-current. For the inlet boundary, certain velocity and temperature of the HTF are considered as the boundary conditions, while outflow condition is considered for the HTF outlet.

**Figure 1:**
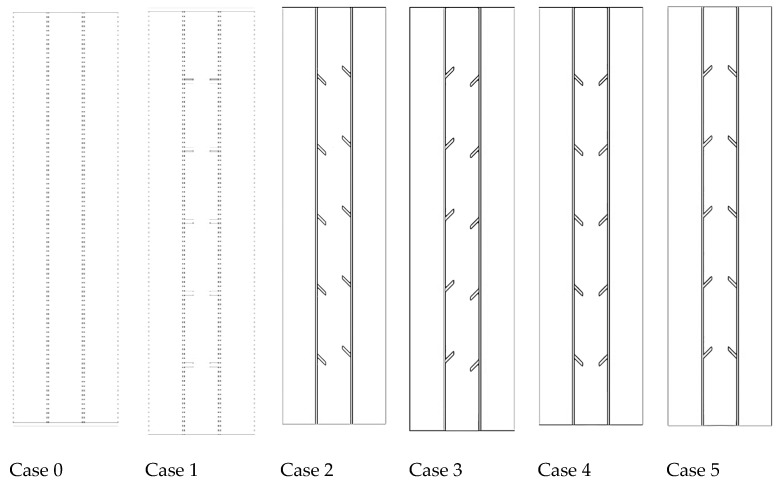
The schematic of different proposed triple-pipe systems in axisymmetric condition; Case 0: no-fin, Case 1: straight fins, Case 2–5: sloped fins with different orientations for the inner and middle tubes.

Due to the nature of the studied problem and lack of circumferential variation in the flow, the system is considered an axisymmetric situation for different studied cases (shown in Figure 1). As shown, five fins are connected to the inner and outer tubes (the total fin number is 10). Four different cases are investigated for the sloped fins, considering different directions for the fins, as shown in Figure 1. Note that the slope of the fins is 45°. The fins dimensions are 2 mm thickness and 5 mm length for the systems with smooth fins. The dimensions of the fins in the sloped fin cases are determined to have the same area compared with the smooth fin case. Furthermore, the mass of the PCM is similar in the fin cases as well as the finless case. Note that the distances between the fins are considered 40 mm to uniformly be distributed in the domain.

**Figure 2:**
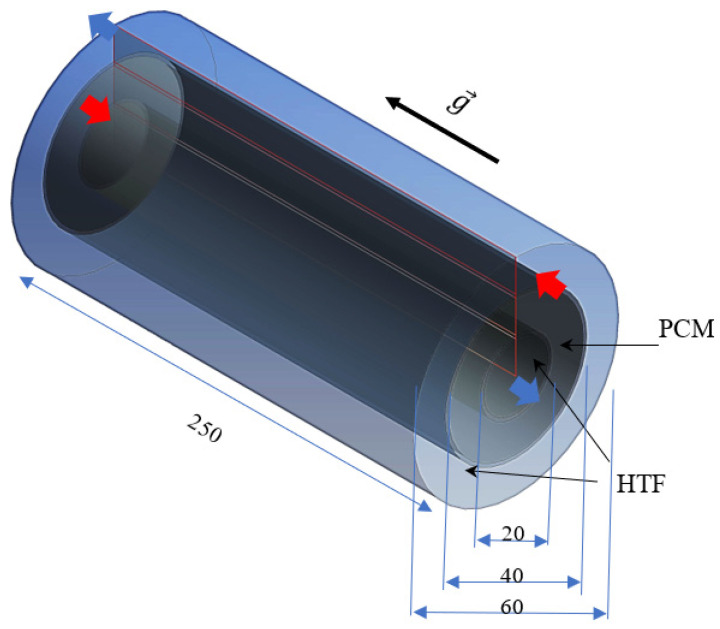
The schematic of the studied vertical triple-tube heat exchanger (all the dimensions are in mm).

The schematic of the system in 3D is illustrated in Figure 2 for the finless case, considering all the dimensions, gravity direction, and fluid flow directions.

The inlet temperature and Reynolds number for the HTF are 50 °C and 1000, respectively, to find the best configuration for the fins. In addition, as a sensitivity analysis for the fin’s dimensions of the best system, the inlet temperature and Reynolds number of the HTF are also studied for the best case. The initial temperature of the PCM is also considered 15 °C.

## 3. Mathematical Modeling

The simulation of the phase-change process was achieved by applying the enthalpy–porosity method, which was defined by Brent et al. [32,33]. A similar value of the porosity and the liquid fraction for each cell were assumed the activated domain. The natural convection impact in the system was handled based on the Boussinesq approximation. In this approximation, the density of PCM is considered constant in all terms, except for the gravity term in the momentum equation, in which the density is assumed to vary with temperature, according to the following equation:

$$\rho =\rho_{ref}\left(1-\beta \left(T-T_{ref}\right)\right)$$

This approximation is essential for modeling the flow of liquid PCMs during their phase transition. If the momentum equation with no density variation is applied, then the natural convection effect cannot be considered because there is no buoyancy-driven flow generated. Previous research has consistently shown that natural convection plays a vital role in expediting the phase transition of PCMs, particularly during the melting mode. It may also dominate the PCM phase-transition modes in some cases [34,35,36]. 

The main assumptions in this work are the laminar and incompressible flow with the transient and Newtonian phenomenon. The viscous dissipation and heat loss to the ambient are also ignored [37,38]. With these assumptions, the conservation equations of continuity, momentum, and energy are then given as [39]:

(1) $$\frac{\partial \rho }{\partial t}+\nabla .\rho \vec{V}=0$$

(2) $$\rho \frac{\partial \vec{V}}{\partial t}+\rho \left(\vec{V}.\nabla \right)\vec{V}=-\nabla P+\mu \left(\nabla^{2}\vec{V}\right)-\rho_{ref}\beta \left(T-T_{ref}\right)\vec{g}-\vec{S}$$

(3) $$\frac{\rho C_{p}\partial T}{\partial t}+\nabla \left(\rho C_{p}\vec{V}T\right)=\nabla \left(k\nabla T\right)-S_{L}$$

The last term in the momentum equation ( $\vec{S})$ is included because of the effect of the melting process, which is the damping term of Darcy law, described as [40]:

(4) $$\vec{S}=A_{m}\frac{{\left(1-\lambda \right)}^{2}}{\lambda^{3}+0.001}\vec{V}$$

where the mushy zone constant $A_{m}$ is considered 10^5^, according to the literature [41,42,43]. For the effect of latent heat and phase-change process in the energy equation, a source term is added, where λ (liquid fraction of PCM) is introduced as [44]:

(5) $$\lambda =\frac{\Delta H}{L_{f}}=\left\{\begin{array}{cc} 0 & if\;T<T_{Solidus}\\ 1 & if\;T>T_{Liquidus}\\ \frac{T-T_{Solidus}}{T_{Liquidus}-T_{Solidus}} & if\;\;\;T_{Solidus}<T<T_{Liquidus} \end{array}\right\}$$

The source term $S_{L}$ in the energy equation is obtained as follows:

(6) $$S_{L}=\frac{\rho \partial \lambda L_{f}}{\partial t}+\rho \nabla \left(\vec{V}\lambda L_{f}\right)$$

The rate of stored energy during the melting process is then defined as:

(7) $$\dot{E}=\frac{E_{e}-E_{i}}{t_{m}}$$

where $t_{m}$ is the melting time, and $E_{e}$ and $E_{i}$ are the total energy of the PCM at the end and beginning of the melting process. E is the summation of sensible heat $\left(MC_{p}dT\right)$ and latent heat $\left(ML_{f}\right)$ of the PCM.

The governing equations for the heat-transfer fluid flow are the conventional Navier–Stokes equations, where the effect of phase change is eliminated from Equations (1)–(3). The HTF flow is laminar in this study.

Paraffin RT-35 is utilized as the PCM with thermophysical properties listed in Table 1. Basically, there are several impacts that could nullify the operation of the heat energy storage, such as the limited energy density, material compatibility, properties variation during the phase-change process, corrosion, phase separation, lack of thermal stability, and inflammability [45].

## 4. Numerical Model

Ansys fluent software is utilized to solve the governing equation of the system. The model uses the SIMPLE algorithm for the pressure–velocity coupling and the Green–Gauss cell-based method for computing the variables’ gradients. Likewise, the QUICK differencing scheme is used to solve the momentum and energy equations, while pressure correction equations are used by adopting the PRESTO scheme. The under-relaxation factors are considered to be 0.3, 0.3, 0.5, and 1 for pressure correction, velocity components, liquid fraction, and energy equation, respectively. The convergence criteria for the continuity, momentum, and energy equations are set to be 10^−4^,10^−4^, and 10^−6^, respectively.

**Figure 3:**
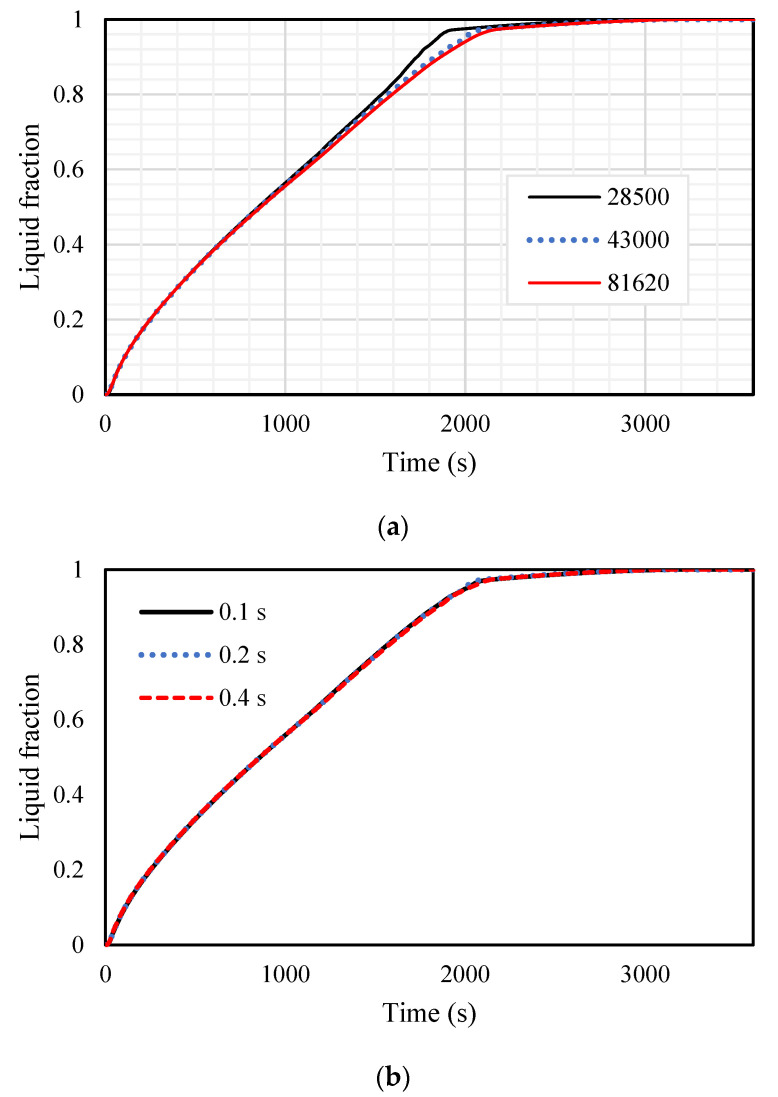
The variation of liquid fraction for different (**a**) sizes of the gird and (**b**) size of time step.

The grid independence analysis, as well as time step size analysis, were investigated. For the mesh independency analysis, different sizes of the mesh from 28,500, 430,000, and 81,620 for the number of cells are evaluated using the time step size of 0.2 s for the inline-finned case with uniform fins arrangement. The results are displayed in Figure 3a and are almost identical for the grid sizes of 43,000 and 81,620, and therefore, the mesh size of 43,000 is chosen for further analysis. Different sizes of 0.1, 0.2, and 0.4 s for the time step size for the selected grid are also investigated, shown in Figure 3b, and 0.2 s is selected for the size of the time step. The number of iterations was less than 100 for every time step at the beginning of the melting and solidification processes to meet the convergence criteria.

**Figure 4:**
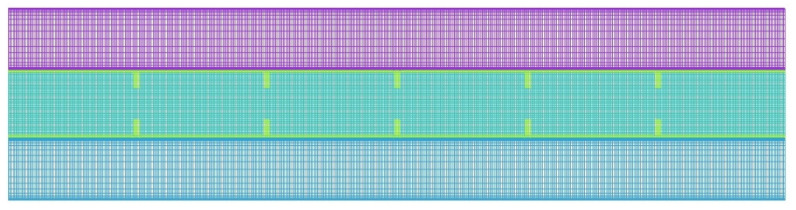
The configuration of the mesh after grid independence analysis (using 43,000 cells).

The mesh with the grid numbers of 43,000 is illustrated in Figure 4.

**Figure 5:**
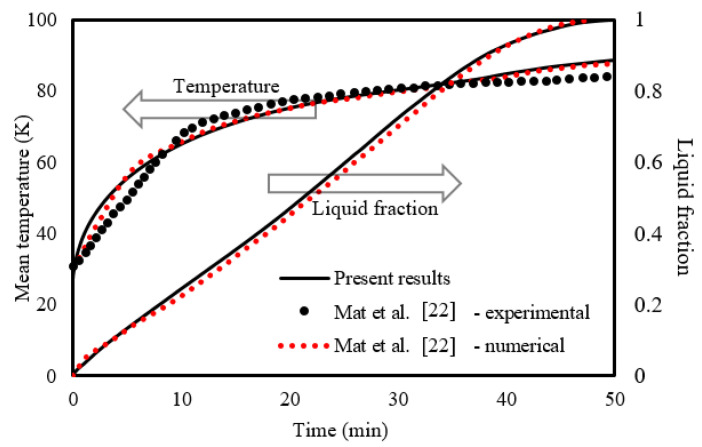
Verification of the numerical model.

The numerical model is tested using the experimental and numerical outcomes of Mat et al. [41], where the influence of fins attached to both the outer and inner wall of the channel in the PCM (RT58) area in a double-tube LHSHE system was studied. As seen from Figure 5, the presented results are in line with the experimental results for the temperature and numerical results for the liquid fraction of Mat et al. [41].

## 5. Results and Discussion

This section explains the effects of the fin’s distortion direction (orientation) and the fin’s size. Six different cases are considered, as shown previously in Figure 1. In addition to the no-fin case, different orientations of the fins are included in this study. Then, the performances of the finless system are compared with different fins’ orientation systems, and the best case regarding the performance of the heat transfer is selected. The final investigation includes the impacts of the heat-transfer fluid (water) temperature and velocity, which is represented by the Reynold number.

### 5.1. Effect of Various Orientations of the Fins

Adding fins to the TES improves the efficiency of the unit because of enlarging the surface area of the heat transfer and increasing the average thermal conductivity of the unit, as the thermal conductivity of the metal (fins) is higher than the PCM itself. Adding fins also delivers heat (from the outer wall (right-hand side of the contours) and the inner wall (left-hand side of the contours)) to the deep region of the PCM, causing a faster melting process. Natural convection is strongly affected by the fins during the melting process, as the fins form a barrier in the molten PCM direction. Five fins (5 mm long and 2 mm thick) are merged to the shell part of the system. 

**Figure 6:**
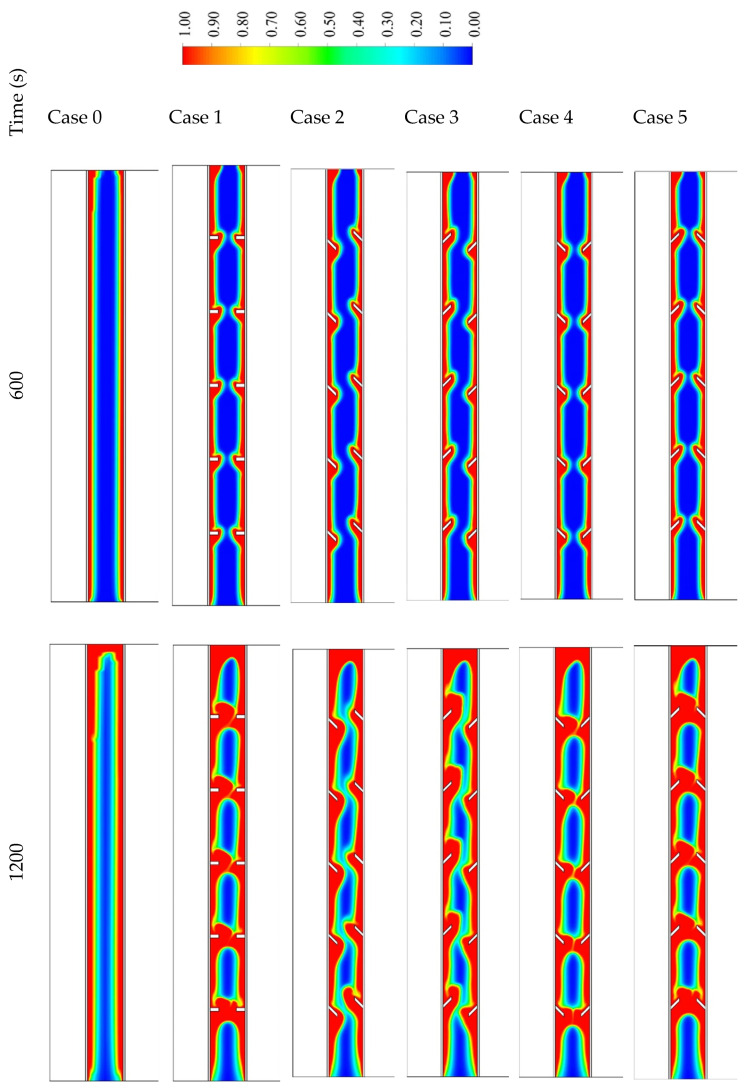

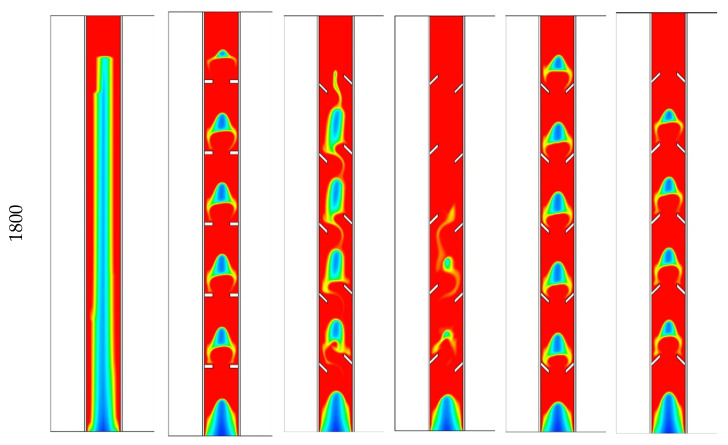
The liquid fraction development during 1800 s in three different time steps for the case of finless and the cases of different orientations of fins directions. Note that the right- and left-hand-side walls are the outer and the inner walls, respectively.

Six different cases are considered in this study, as shown in Figure 6. Case 0 presents the system without fins, case 1 includes five opposite fins in a horizontal direction (inline) on each side, and case 2 has distorted fins downward on the left side and distorted fins upward in the right side of the shell. The orientations of the fins in case 3 are opposite of those in case 2. The fins in case 4 are all directed downward, and those in case 5 are directed upwards. Figure 6 shows the generation of the liquid fraction for all the cases in three time steps, 600, 1200, and 1800 s. In finless cases, the PCM melts initially at the attached area to the HTF walls. Over time, extra PCM liquefies, creating a thicker liquid layer at the wall and collecting at the top because of the free convection. The PCM is still solid at the lowest area of the system. In the 1800 s, only 68% of the PCM melts, and this value will increase by applying fins in the unit. Images in the second column of the figure show the inline fin case (case 1). The distance between the fins is 40 mm on each side (the inner wall and the outer wall), with a 40 mm separation space from the upper and lower sides. The molten PCM appears beside the walls and around the fins, and the layer of the generated liquid enlarges over time. A portion of a solid PCM is stuck over every two opposite fins; however, the space between the two opposite fins is entirely melted. Within the 1800 s, the total PCM melts are 89%, and the remaining solid part is split into six pieces. Due to the natural convection, circulation of the liquid PCM generates in the clockwise direction, but the fins act as a barrier of this movement, which limits the free convection. To solve this issue, the orientation of the fins was changed and analyzed to achieve better movement of the molten PCM. 

In case 2, the fins attached to the inner wall are oriented downward, and those on the outer wall are directed upward. This configuration causes an extra barrier to front the liquid PCM flow, as the PCM is restricted in the acute angle between the fin and the wall. The total molten PCM in this case at 1800 s is 85%. Case 3 is the opposite configuration of case 2. The flow faces an obtuse angle on both sides of the domain, causing more fluent flow for the liquid PCM. The heat transfer due to the convection process enhances, causing a faster charging process. In 1800 s, 95% of the PCM melts, which is considered the best case studied. Case 4 has fins in a downward direction. The flow of the liquid PCM faces a sharp (acute angle) barrier, when the fluid rises during the circulation and fluently move down due to the obtuse angle of the fins attached to the outer wall. The reverse behavior is achieved in case 5, with fins oriented upward, the molten PCM flow on the obtuse angle during the rising and through an acute angle during the moving down of the PCM movement. The total molten PCM is 88% in both cases 4 and 5.

**Figure 7:**
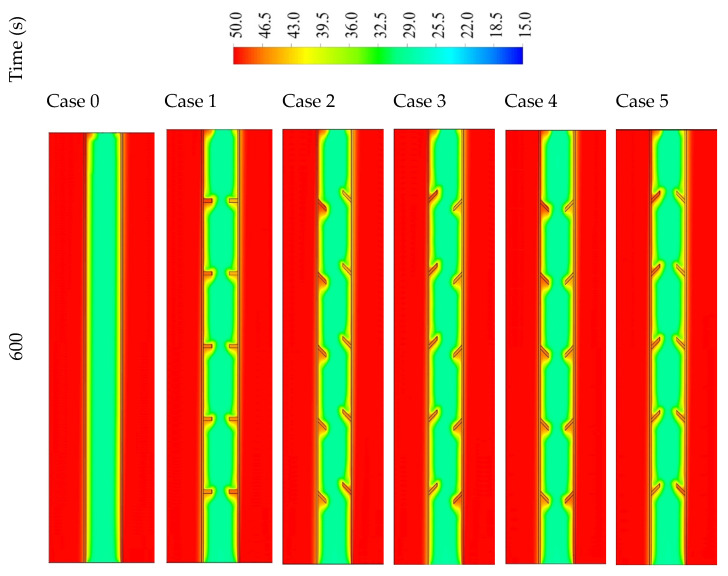

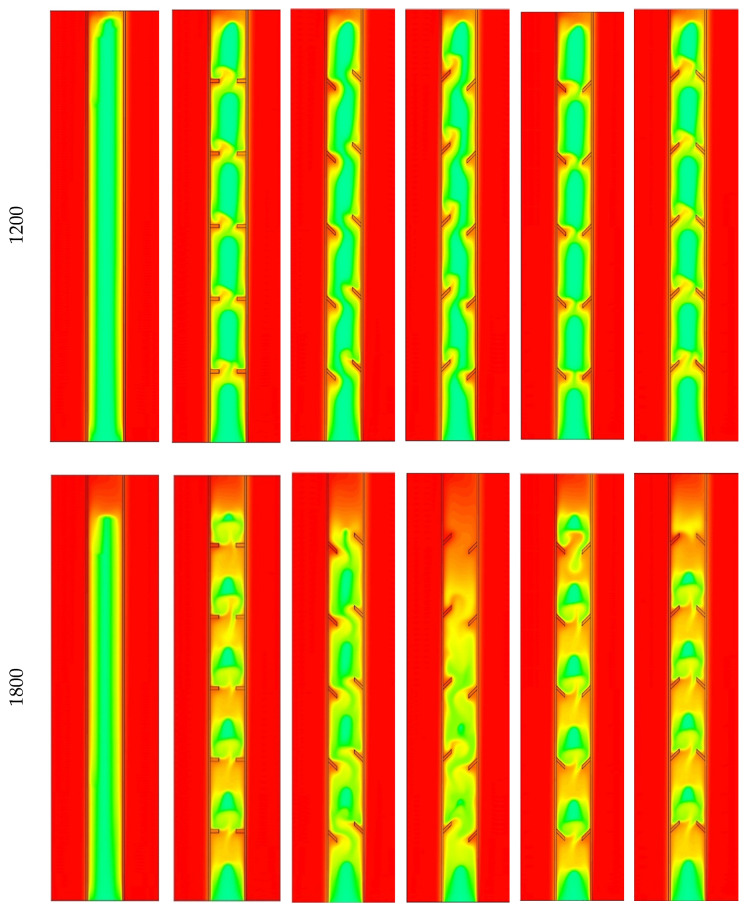
The temperature distribution in 1800 s in three different time steps for the finless case and the cases of different orientations of fins directions.

Figure 7 shows the temperature distribution in 1800 s for the mentioned cases. The channels of the heat-transfer fluid maintain at a fixed temperature (shown in red color) during the charging process due to the short length of the channel. For the finless case, the temperature increases at the attached region to the HTF channel walls. The closer is to the center, the lower the temperature of the PCM registered. Because of the free convection, the generated melted PCM collects at the upper part, which describes the high-temperature area at the upper part of the system. The temperature at the top area arrives at the equilibrium value with the HTF, and over time, the equilibrium region spreads out through the domain. In the 1800 s, only 11% of the PCM reaches the equilibrium value with the HTF temperature. The temperature of the fins practically reaches the temperature of the HTF due to the small size (2 mm × 5 mm) of the utilized fins. As revealed, the fins transfer to the PCM with a uniform distribution. Although the small size of the PCM region has a temperature the same as the HTF temperature compared with the finless case, the mean temperature in the case of inline fins is relatively higher. The temperature of the PCM is affected by the direction of the fins. The fins in case 2 form a strong barrier in front of the circulation of the molten PCM on both sides, causing the lowest heat transfer to the PCM among all the cases with fins; consequently, a lower PCM temperature is registered. Case 3 is considered the best case regarding the PCM circulation among all the utilized fins cases. The higher convection heat-transfer value, causing a higher average temperature of the PCM. Cases 4 and 5 have almost the same average PCM temperature, because in both cases, the fins create a strong barrier (acute angle) at one side and a soft barrier (obtuse angle) at the other side.

**Figure 8:**
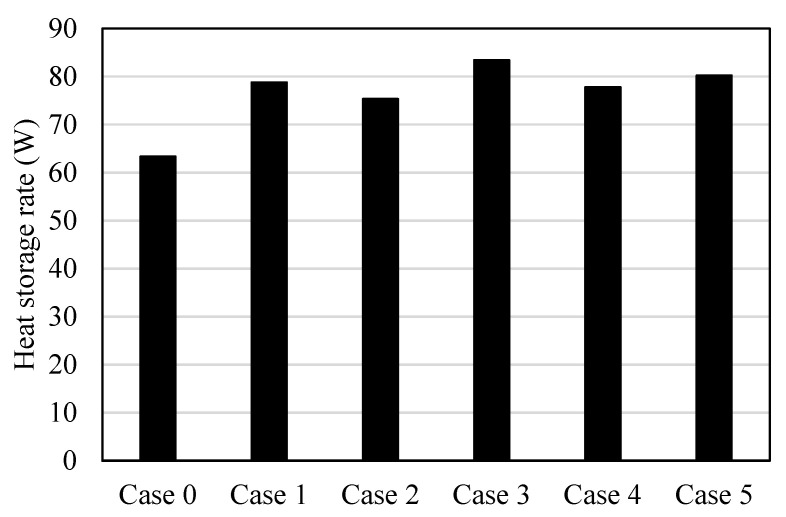
Average heat storage rate at 1800 s of the operation time for the case of finless and the cases of different orientations of fins directions.

Figure 8 shows the heat storage rate for all the cases in 1800 s. The heat storage rate shows the minimum value (64 W) on the finless case because of the limited heat-transfer area, which is provided just by the internal and external HTF walls, and the maximum value (84 W) found for case 3, for the reason explained previously. Case 2 shows the lower heat storage rate among the cases combined with fins due to the barrier of the acute angle fins in front of the PCM flow. Case 1, which has horizontal fins, has exact values between cases 2 and 3, which have opposite directions of fins, and cases 4 and 5, which also have opposite directions of fins.

**Figure 9:**
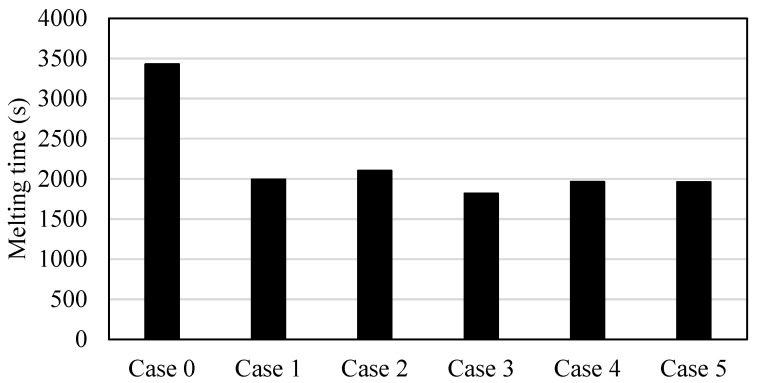
Melting time until reaching 95% of the capacity for the finless case and the cases of different orientations of fins directions.

The behavior of the heat transfer can reflect the melting time. Figure 9 illustrates that the time for melting is only 95% of the PCM. The reasons for choosing 95% are first to guarantee that the melting process is still in the latent heat range (if the melting reaches 100%, the PCM temperature may increase more than the melting point temperature and enter the range of the sensible heat, which is not desired). Further, passing the limits of 95% takes a long time to reach the total melting state. The shortest melting time found in case 3 is due to the highest effect of the natural convection (besides the conductive heat transfer), which is caused by the fluent flow of the liquid PCM over the wall and the fins. The melting time in case 3 is shorter than the melting time of cases 0, 1, 2, 4, and 5 by 88%, 10%, 18%, 8%, and 8%, respectively. The longest melting time was found in the finless case, which is about double that for the best case (case 3).

**Figure 10:**
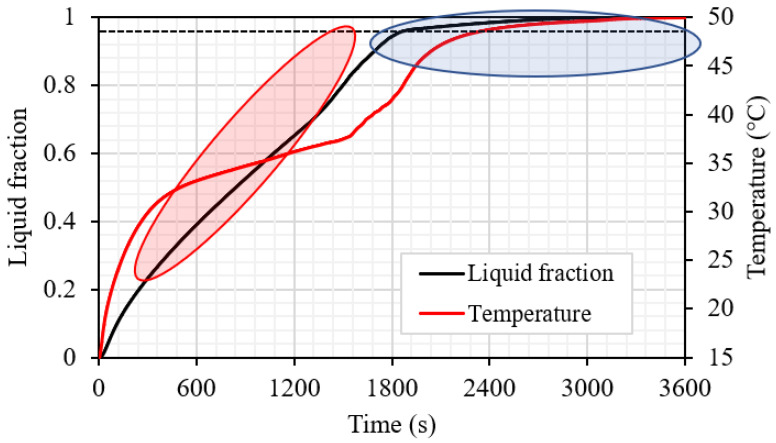
Liquid fraction and mean temperature profiles for the best case (case 3), which is dependent on the next steps of this study.

Figure 10 shows the liquid volume fraction and the temperature development for case 3. The liquid fraction profile shows a linear relationship until the limit of 95%, which takes 1818 s. However, the total melting time reaches 3023 s. This is caused by the domination of the natural convection over the conduction heat transfer, which is limited due to the almost disappearing solid part in the domain. The temperature line shows two changes, the first change when the melting time reaches 50% and the other change when the melting time reaches 95%. 

### 5.2. Effect of Fins’ Size Considering Constant Volume for the Fins

**Figure 11:**
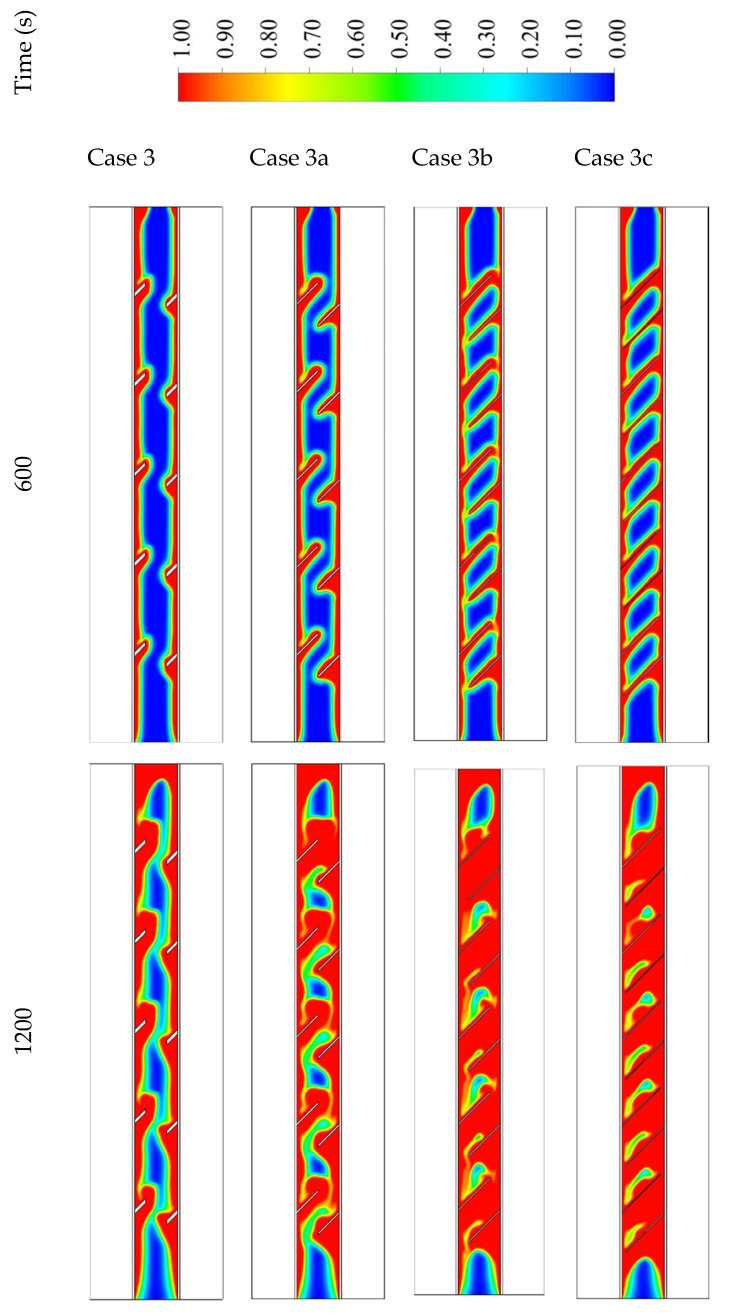

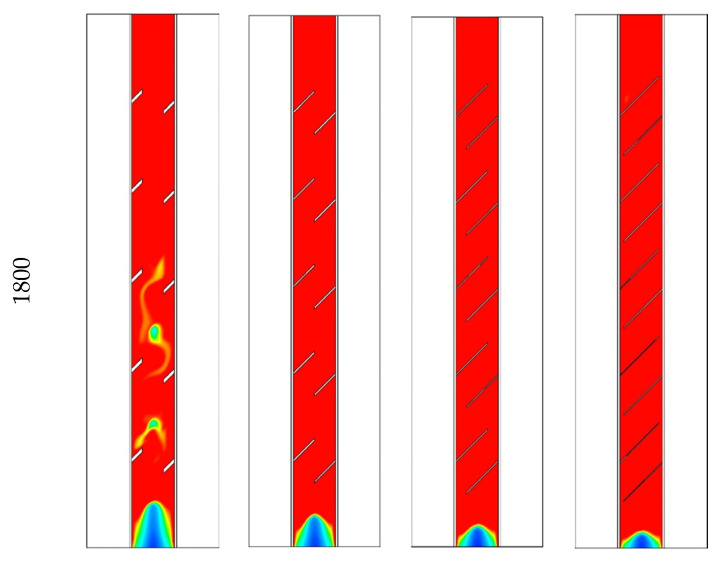
The liquid fraction development in 1800 s in three time steps for different dimensions including case 3 (2 × 7.071 mm^2^), case 3a (1 × 14.14 mm^2^), case 3b (0.666 × 21.21 mm^2^), and case 3c (0.55 × 25.76 mm^2^).

The influence of the fins’ size is analyzed considering the best case among the studied cases regarding the melting time and melting rate, which was case 3 (the fins attached to the inner wall directed upward and those attached to the outer wall directed downward). The dimensions studied include case 3 (2 × 7.071 mm^2^), case 3a (1 × 14.14 mm^2^), case 3b (0.666 × 21.21 mm^2^), and case 3c (0.55 × 25.76 mm^2^) (W × L) (shown in Figure 11), taking into account the same sizes of the fins in all the cases. Because of the large surface area of the longer fins, the rate of the heat transfer is higher, causing a quicker melting process, as shown in Figure 11. Although, the longer and thinner fins (0.55 × 25.76 mm^2^) diminish the gap area between the fins’ edge and the opposite wall, which produces a barrier front to the liquid PCM. This limits the heat transfer by the natural convection effect. For the small fins (case 3), the solid phase is shown as a serpentine; over time, the continuous solid state becomes thinner, then producing a scattered small portion of the solid PCM. Within 1800 s, 95% of the PCM is converted to the liquid phase. For case 3a (1 × 14.14 mm^2^), the melting process of the PCM approximately demonstrates the same behavior as the short fins, but at 1200 s, the solid part appears as pieces between the fins. In cases 3b and 3c, the PCM melts around the fins and the wall, and because of the long fins utilized in these cases, the solid part converts to pieces between the fins at the first 600 s. In cases 3b and 3c, the melting process in 1800 s achieves 97.5% and 98%, respectively, which have minor differences with the other two cases. The only different occurrence observed in cases 3b and 3c is the constraint of the PCM between two neighbor fins. This fact restricts the ability of thermal expansion in the PCM area. 

**Figure 12:**
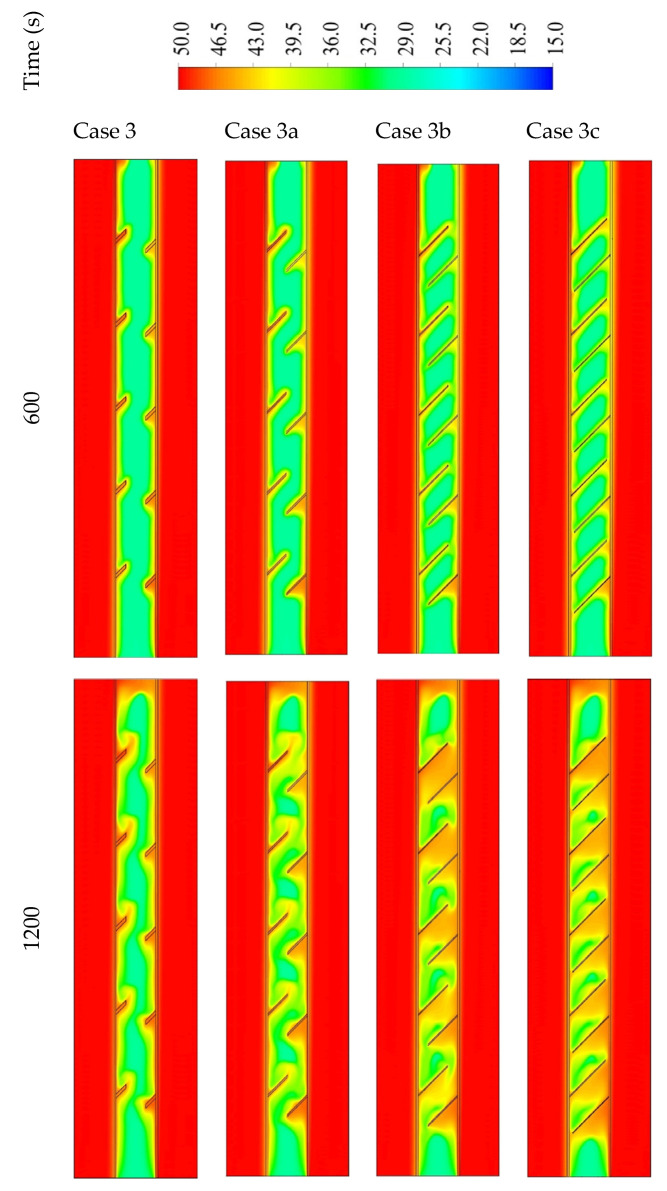

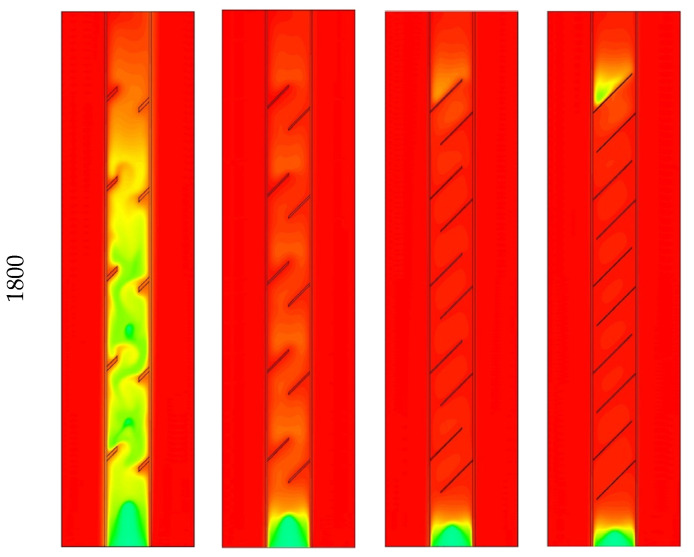
The temperature distribution in 1800 s in three time steps for different dimensions including case 3 (2 × 7.071 mm^2^), case 3a (1 × 14.14 mm^2^), case 3b (0.666 × 21.21 mm^2^), and case 3c (0.55 × 25.76 mm^2^).

Figure 12 shows the temperature distribution in the PCM domain in 1800 s for all the cases. With the short fins (case 3), the temperature distributes uniformly but in slow steps, as the heat could not be transferred to the deep part of the PCM. The warmer PCM gathers at the top, and the cold part collects at the bottom; this is caused by the large free space in the short fin case helping the liquid PCM to move better due to the natural convection. Within the 1800 s, the average temperature reaches 41.5 °C in the whole domain. Using longer fins (case 3a (1 × 14.14 mm^2^)) increases the temperature generally through all the units because of the large surface area of the heat transfer, which is provided by the longer fins. In this case, the space of the movement reduces relatively compared with the short fins. The mean temperature in the unit reaches 48 °C in 1800 s. For case 3b (0.666 × 21.21 mm^2^), as stated earlier, the PCM restricted the area between the two neighbor fins with a narrow space between the fin and the opposite wall. The heat spread faster because of the larger surface area of the fins; although, the dimensions of the fins limit the movement over the whole unit. On the other hand, a movement is created in the region between the two adjacent fins. The mean temperature in the whole system is 47.4 °C and 47.6 °C in cases 3b and 3c, respectively. Generally, most of the regions between the fins nearly reach the thermal equilibrium with the HTF temperature. However, for the higher region, the PCM obtains heat from the lower fin, and the upper fins are missing because of the wall, which causes a remaining solid portion at the top section of the domain.

**Figure 13:**
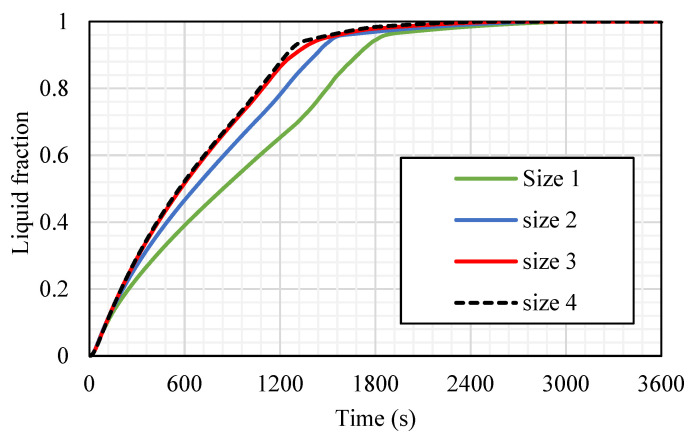
The liquid fraction development for the total PCM for the cases (case 3 (size 1: 2 × 7.071 mm^2^), case 3a (size 2: 1 × 14.14 mm^2^), case 3b (size 3: 0.666 × 21.21 mm^2^), and case 3c (size 4: 0.55 × 25.76 mm^2^)).

Figure 13 shows the liquid fraction progress for the four cases in 3600 s. For case 3, the liquid fraction develops at a constant rate until 1818 s (with 95% molten PCM), then due to the natural convection effect, the heat-transfer rate reduces, and total melting is achieved within 2900 s, causing more uniform temperature distribution. The curve for case 3a shows a constant growth rate of the liquid fraction until 1533 s (with 95% of molten PCM); then, the liquid generation rate reduces, and the total melting time is 2700 s. For the longer fins in cases 3b and 3c, the procedures of the melting process are the same, and both reach the entire melting level faster than cases 3 and 3a; but indeed, as explained previously, the case with the longest fins (case 3c) has a slightly faster melting time than case 3b.

**Figure 14:**
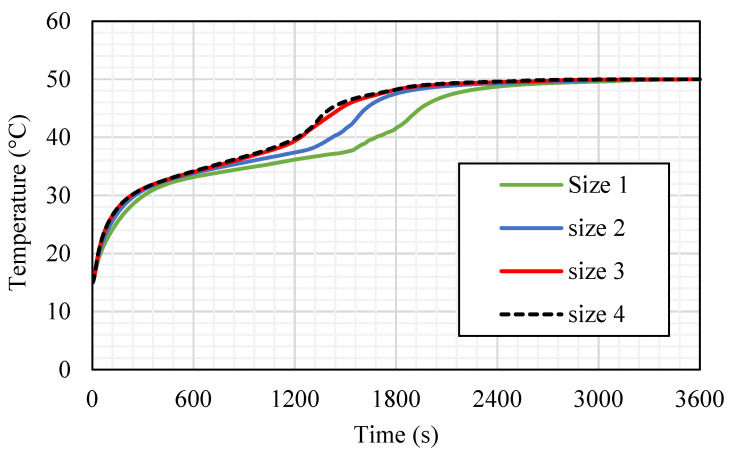
The average temperature profiles for the total PCM for the cases (case 3 (size 1: 2 × 7.071 mm^2^), case 3a (size 2: 1 × 14.14 mm^2^), case 3b (size 3: 0.666 × 21.21 mm^2^), and case 3c (size 4: 0.55 × 25.76 mm^2^)).

The mean temperature noticeably rises with the first 200 s because of the conduction effect, as shown in Figure 14. The free convection forming in the molten PCM closes the walls, and the fins cause a reduction in the temperature rising rate. The case with the longest fins (3c) has a higher average temperature value because of the large surface area. Table 2 shows that the melting time reduces by 15.6%, 19.2%, and 22% with the fins of cases 3a, 3b, and 3c, respectively, compared with case 3. The charging rate improves by 7.8%, 9.2%, and 9.6% with the fin cases 3a, 3b, and 3c, respectively, compared with case 3. The summary of this section revealed that the longer fin provides a higher surface area of heat transfer, which means a quicker charging procedure and higher melting rate. However, since there is a tiny difference between these parameters in cases 3b and 3c, case 3b is chosen as the best length regarding the heat-transfer and melting time. Accordingly, case 3b is used to evaluate the effects of the Re and the inlet temperature of the HTF.

### 5.3. Effects of HTF Reynolds Number

**Figure 15:**
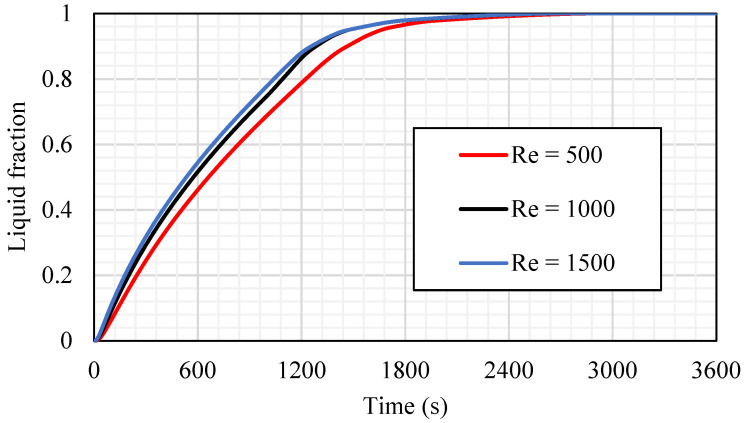
The liquid fraction development for the total PCM for case 3b (Tin equals 50 °C) at different values of Re (500, 1000, and 1500).

The influence of the Reynold number (Re), which presents the flow rate of the HTF, is investigated in case 3b (fin size equals 0.666 × 21.21 mm^2^), as shown in Figure 15. Three different values of the laminar flow Re (500, 1000, and 1500) have been considered in this study. The higher value of the Re provides a faster melting process, as the faster flow of the fluid increases the convection heat transfer to the HTF channel wall, then to the PCM. The effect of the Re appears in the constant gradient and fix evaluation until the molten PCM reaches 95% (at 1659 s for Re equal to 500 and around 1470 s for Re equal to 1000 and 1500). After those times, the gradient slows down until the whole PCM melts. It is worth mentioning that the effect of both higher Res (1000 and 1500) are quite similar regarding the melting time, PCM mean temperature, and the heat-transfer rate from the HTF to the PCM. Therefore, the best value of Re that should be considered is 1000.

**Figure 16:**
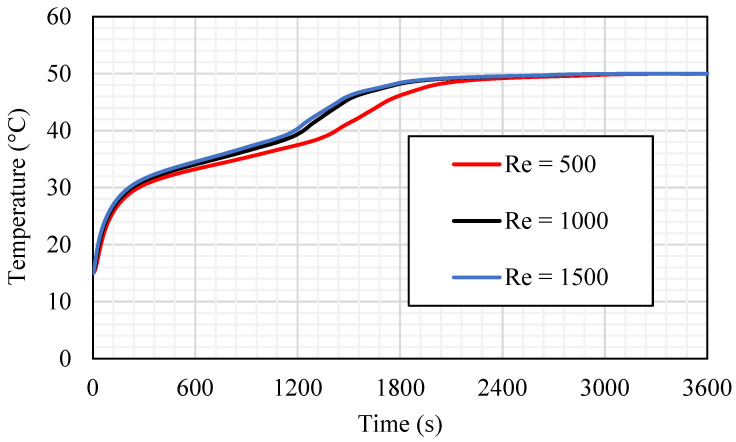
The average temperature profile for the total PCM for the case of 3b (Tin equals 50 °C) at different values of Re (500, 1000, and 1500).

In the thermal profile analysis (shown in Figure 16), the mean temperature line for the case of Re equals 500 shows a minor performance compared with the other cases. Within 2400 s of the system operation time, the PCM reaches a stable value of 50 °C (equilibrium with the HTF temperature); whereas, for the cases of Re equals 1000 and 1500, the average temperature of the PCM reached the stable value (50 °C) within the same time, which is about 1800 s. The gradient of the temperature line is varying clearly at 1200 s when Re equals 1000 and 1500 and at 1500 s when the Re is 500 due to the melting process, which develops a thermal convection impact. The difference in the average PCM temperature for the cases of Re equals 1000 and 1500 is very small, which can be ignored. 

Table 3 shows the melting time for the 95% of the PCM and the heat-transfer rate in the first 1800 s for the mentioned values of Re. The melting time and the heat-transfer rate for the cases of Re equals 1000 and 1500 are quite similar. The differences between melting time and the heat-transfer rate for cases of Re equals 1000 and 1500 are 0.06% and 0.17%, respectively. However, the melting time is reduced by 11.4%, and the heat-transfer rate increases by 2.9% compared with those for Re equals 500. Because of the tiny differences between the TES performance in cases of Re equals 1000 and 1500, the best value of Re chosen for the further study is 1000.

### 5.4. Effect of HTF Inlet Temperature

**Figure 17:**
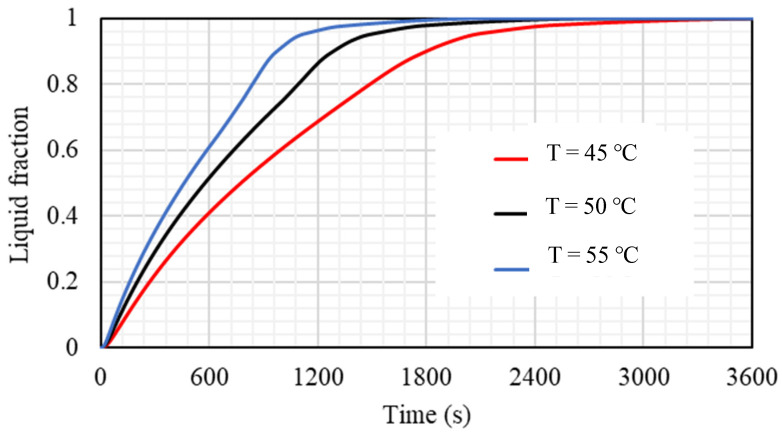
The liquid fraction development for the total PCM for the case 3b (Re equals 1000) at different values of inlet HTF temperature (Tin = 45 °C, Tin = 50 °C, and Tin = 55 °C).

The inlet temperature of the HTF has a great influence on the performance of the TES, and it is investigated in this section. Case 3b (0.666 × 21.21 mm^2^) with Re equal to 1000 is used to evaluate the effects of three different nominated values (45, 50, and 55 °C) of TES inlet temperature. Figure 17 illustrates that the liquid fraction is considerably affected by changing the thermal condition of the HTF, and the melting rate is proportional to the inlet temperature. A higher inlet temperature produces the high-temperature difference between the bulk and the wall; accordingly, more heat moves from the HTF to the PCM, causing a quicker melting procedure. The times required to melt 95% of the PCM are 1101 s, 1469 s, and 2049 s when the inlet temperatures are 45, 50, and 55 °C, respectively. 

**Figure 18:**
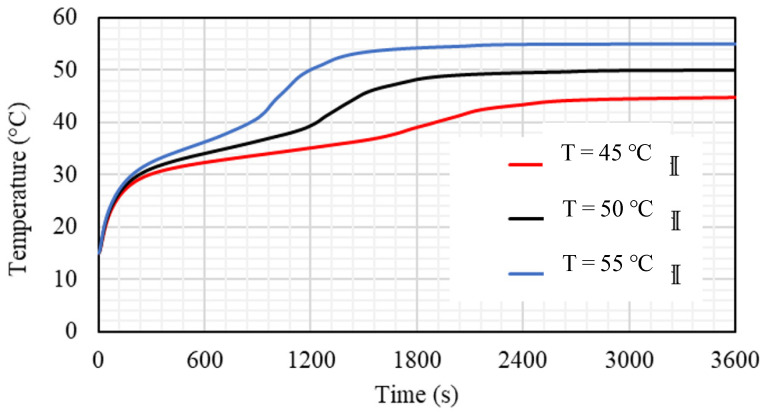
The average temperature of the PCM for the case 3b (Re equals 1000) at different values of inlet HTF temperature (Tin = 45 °C, Tin = 50 °C, and Tin = 55 °C).

The mean temperature of the PCM is directly affected by the inlet temperature of the HTF; whereas, the average temperature of the PCM achieves a higher value by applying the highest temperature of the inlet HTF. The PCM reaches a higher temperature utilizing the warmer HTF and achieves the thermal equilibrium with the HTF earlier. The average temperature of the PCM rises sharply in the first 200 s because of the conduction heat transfer, as most parts of the PCM are still solid, then the gradients of the lines are reduced due to the melting process and increasing the impact of the convection process on account of the conduction heat. At 1800 s of the operation time, the mean temperature of the PCM reaches 39.1, 48.3, and 54.3°C when the inlet temperatures are 45, 50, and 55 °C, respectively (as shown in Figure 18). The system reaches the thermal equilibrium with the HTF temperature at 2200 s, 2400 s, and 3000 s, when the inlet temperatures used are 45, 50, and 55 °C, respectively. This action is caused by a higher temperature difference, which causes more thermal exchange between the HTF and PCM and results in reaching thermal equilibrium faster in the case of the highest HTF temperature. 

Table 4 shows the impact of the inlet HTF temperature on the performance of the TES, which is presented by the melting time of 95% of the PCM melting and the heat-transfer rate in 1800 s. As mentioned previously, the melting time reduces with increasing inlet HTF temperature. The duration of the melting reduces by 28% and 46%, when the inlet temperature increases from 45 °C to 50 °C and 55 °C, respectively. The heat-transfer rate is proportional to the inlet HTF temperature; whereas, the thermal rate increases by 16% and 23.6% when the temperature increases from 45 °C to 50 °C and 55 °C.

## 6. Conclusions

The high thermal resistance of PCMs, which alters the heat charging/discharging response rates of these materials, has been identified as the fundamental factor limiting their effectiveness as heat storage media. This necessitates the introduction of further enhancement additives, such as extended surfaces and fins, to enable the entire PCM to be heat charged and discharged with minimal thermal resistance effects. Circular fins of various sizes and orientations were suggested in this work for modifying the low thermal response rates of PCM during the melting mode in a triple-tube heat exchanger TES containment. A series of simulation studies for PCM melting were carried out and reported, using varied fin designs, fin dimensions, fin orientations, as well as different thermal and flow boundary conditions of the heat-transfer fluid (HTF). In terms of local temperature distribution, liquid fraction evolution, melting time, and charging power, the potential of the aforementioned parameters on improving the melting enhancement rates of PCM was theoretically analyzed and assessed.

The fins were directed as downward–downward, upward–upward, and downward–upward along the left side and the right side of the shell to reveal the impact of fin orientation. The downward–upward fin orientation was the best-performing case in terms of the melting rate and the heat storage rates. The impact of varying the fin dimensions (i.e., fin thickness and height) considering the same total fin volume fraction and the optimum fin orientation was investigated at fin sizes of (2 × 7.071 mm^2^), (1 × 14.14 mm^2^), (0.666 × 21.21 mm^2^), and (0.55 × 25.76 mm^2^). The latter case provided the best melting time reduction by 22% and the highest heat charging rate by 9.6% compared with the base case of 2 × 7.071 mm^2^ fin size. Different values of Re 500, 1000, and 1500 were considered, and it was found that the melting time for the case of Re = 1500 was reduced by 11.4%, and the heat-transfer rate increased by 2.9% compared with those for the case of Re = 500. Finally, the results confirmed that the mean temperature of the PCM is directly affected by the inlet temperature of the HTF; whereas, the average temperature of the PCM achieves a higher value by applying the highest temperature of the inlet HTF. It was found that increasing the inlet HTF temperature from 45 to 55 °C reduces the duration for the melting completion by 46%. It is worthy to mention that the concept of using sloped fins with different sizes and orientations in PCM-based storage systems has not been examined before. Numerical predictions from this study reveal an encouraging improvement in storage performance when these techniques are used. Further, some additional ideas including the consideration of the heat-transfer rate and the pressure drop can be recommended to be implemented in the future.

## Figures and Tables

**Table 1:** Thermo-physical properties of Paraffin R-T35 [46].

Properties	ρl [kg/m^3^]	$\rho s_{\;}[\mathrm{kg}/m^{3}]$	L_f_ [kJ/kg]	C_p_ [kJ/kg.K]	K [W/m.K]	µ [N.s/m^2^]	T_L_ [°C]	T_S_ [°C]	β [J/K]
Values	770	860	170	2	0.2	0.023	36	29	0.0006

**Table 2:** Melting time until achieving 95% and the heat-transfer rate in 1800 s for the cases of case 3 (2 × 7.071 mm^2^), case 3a (1 × 14.14 mm^2^), case 3b (0.666 × 21.21 mm^2^), and case 3c (0.55 × 25.76 mm^2^).

	Melting Time until Reaching 95% Capacity (s)	Heat-Transfer Rate during the First 1800 s
Case 3 (2 × 7.071 mm^2^)	1818	83.41151
Case 3a (1 × 14.14 mm^2^)	1533	89.8931
Case 3b (0.666 × 21.21 mm^2^)	1469	91.0916
Case 3c (0.55 × 25.76 mm^2^)	1417	91.41624

**Table 3:** Melting time until achieving 95% and the heat-transfer rate in 1800 s for the cases of Re equals 500, 1000, and 1500.

	Melting Time until Reaching 95% Capacity (s)	Heat-Transfer Rate during the First 1800 s
Re 500	1659	88.56
Re 1000	1469	91.09
Re 1500	1470	91.25

**Table 4:** Melting time until achieving 95% and the heat-transfer rate in 1800 s for the cases of inlet HTF temperature of Tin = 45 °C, Tin = 50 °C, and Tin = 55 °C.

	Melting Time until Reaching 95% Capacity (s)	Heat-Transfer Rate in the First 1800 s
Tin = 45 °C	2049	78.50
Tin = 50 °C	1469	91.09
Tin = 55 °C	1101	97.04

## Nomenclature

*A_m_*	The mushy zone constant (kg/s m^3^)	Greek symbols	
*C*	Inertial coefficient	β	Thermal expansion coefficient (1/K)
$C_{p}$	PCM specific heat (J/kgK)	λ	Liquid fraction
*D*	Hydraulic diameter (m)	α	Thermal diffusivity (m^2^/s)
g	Gravitational acceleration (m/s^2^)	μ	Dynamic viscosity (kg/ms)
K	Thermal conductivity (W/mK)	ρ	Density (kg/m^3^)
L	Latent heat of fusion (J/kg)		
m	PCM mass (kg)	Subscribe	
P	Pressure (Pa)	ref	Reference
$\dot{Q}$	Solidification rate (W)	l	Liquid
$t_{m}$	Solidification time (s)	s	Solid
T	Temperature (K)	f	Fraction
$T_{m}$	Melting point temperature (K)	m	Mean
$u_{i}$	Velocity component (m/s)	e	Final
$\vec{V}$	Velocity vector (m/s)	i	Initial
